# Organic heterojunctions: Contact-induced molecular reorientation, interface states, and charge re-distribution

**DOI:** 10.1038/srep21291

**Published:** 2016-02-18

**Authors:** Andreas Opitz, Andreas Wilke, Patrick Amsalem, Martin Oehzelt, Ralf-Peter Blum, Jürgen P. Rabe, Toshiko Mizokuro, Ulrich Hörmann, Rickard Hansson, Ellen Moons, Norbert Koch

**Affiliations:** 1 Institut für Physik & IRIS Adlershof, Humboldt-Universität zu Berlin, Berlin, Germany; 2 Helmholtz-Zentrum Berlin für Materialien und Energie GmbH, Bereich Erneuerbare Energien, Berlin, Germany; 3 National Institute of Advanced Industrial Science and Technology, Osaka, Japan; 4 Institute of Physics, University of Augsburg, Augsburg, Germany; 5 Department of Engineering and Physics, Karlstad University, Karlstad, Sweden

## Abstract

We reveal the rather complex interplay of contact-induced re-orientation and interfacial electronic structure – in the presence of Fermi-level pinning – at prototypical molecular heterojunctions comprising copper phthalocyanine (H16CuPc) and its perfluorinated analogue (F16CuPc), by employing ultraviolet photoelectron and X-ray absorption spectroscopy. For both layer sequences, we find that Fermi-level (E_F_) pinning of the first layer on the conductive polymer substrate modifies the work function encountered by the second layer such that it also becomes E_F_-pinned, however, at the interface towards the first molecular layer. This results in a charge transfer accompanied by a sheet charge density at the organic/organic interface. While molecules in the bulk of the films exhibit upright orientation, contact formation at the heterojunction results in an interfacial bilayer with lying and co-facial orientation. This interfacial layer is not E_F_-pinned, but provides for an additional density of states at the interface that is not present in the bulk. With reliable knowledge of the organic heterojunction’s electronic structure we can explain the poor performance of these in photovoltaic cells as well as their valuable function as charge generation layer in electronic devices.

The understanding of electronic properties of organic semiconductor heterojunctions has grown significantly over the past years. Starting from the simplest approach to predict the energy levels at electrode/organic and organic/organic heterojunctions, i.e., a constant electrostatic potential throughout the structure – often referred to as vacuum level alignment (Fig. 1a), it rapidly transpired that this situation is rather an exception at real interfaces^1^,^2^,^3^,^4^. Numerous physico-chemical processes were identified that affect the energy level alignment. At electrode/organic interfaces the most important examples include the push-back-effect^1^,^5^,^6^,^7^, chemical reactions^8^,^9^,^10^, polarization^11^,^12^, structural imperfections^13^, and contact-induced molecular conformation changes^14^. For ordered molecular assemblies also the molecular orientation with respect to the substrate is important as the ionization energy and electron affinity are orientation-dependent parameters^15^. For weakly interacting (physisorptive) interfaces, Fermi-level (E_F_) pinning was identified as the dominant mechanism that gives rise to level alignment scenarios involving substantial electrostatic potential changes across the interface^1^,^16^. In the E_F_-pinning regime, integer charges (due to the absence of strong electronic coupling) are transferred between electrode and the organic frontier levels because the ionization (affinity) levels of the semiconductor are above (below) E_F_ of the electrode before contact. Consequently, energy level bending (band bending) within the organic layer close to the interface arises^4^,^17^,^18^. The work function at which E_F_-pinning is observed, notably several 100 meV lower/higher than the ionization/affinity levels of the organic semiconductor, was attributed to the energy-relaxed levels of polarons (formed due to the charge transfer) in the gap of the neutral molecular solid^1^,^16^,^19^,^20^. However, more factors contribute to the actual pinning level, which is not an intrinsic material constant. It was shown that the density of states of the organic semiconductor, particularly including gap states that result from chemical defects and structural disorder in the solid, critically influences the pinning position of E_F_^21^,^22^,^23^. In addition, on-site and inter-molecular Coulomb interactions determine where the polaronic (i.e., molecular ion) levels lie with respect to the neutral surrounding organic material^24^. Finally, the amount of charge transferred to reach electronic equilibrium at a given interface directly impacts the position of E_F_ in the gap of the semiconductor. The associated energy level (band bending) leads to gradual further changes of the electrostatic potential away from the interface, which is particularly important for organic semiconductors with very long Debey lengths^17^.

This type of integer charge transfer, due to E_F_-pinning, to reach electronic equilibrium notably also occurs when an insulator is placed between electrode and organic, i.e., the analogue of a metal-insulator-semiconductor (MIS) structure^4^,^24^,^25^ as depicted in Fig. 1b,c. Due to the absence of charge carriers in the “I” layer, the electrostatic potential changes linearly between the electrode and the pinned semiconductor. Most of these processes are likewise relevant for organic semiconductor heterojunctions, which are particularly important in photovoltaic and light emitting applications. However, reports in literature about the energy level alignment at organic heterojunctions yield a picture that is less converged as compared to the one we have for electrode/organic junctions. While, indeed, vacuum level alignment sometimes was reported^1^,^26^, also interface dipoles^1^,^4^,^5^,^26^,^27^ (of debated origin) and energy level bending^4^,^23^,^28^ were postulated to explain photoemission data obtained from such interfaces. Notably, different energy level scenarios were found even for the same material pair^29^,^30^,^31^,^32^, revealing that improved understanding of the electronic processes at organic/organic interfaces is needed^33^.

A material pair of interest for applications of an organic heterojunction is hydrogen-terminated copper phthalocyanine (H16CuPc) in combination with its perfluorinated analogue (F16CuPc), for chemical structures see Fig. 2. For instance, ambipolar transport was found for H16CuPc/ F16CuPc bilayers in field-effect transistors^34^. This was explained by the presence of holes in H16CuPc and electrons in F16CuPc at the interface^35^,^36^. Soon after, planar and bulk heterojunctions were examined in diode structures^37^. Such structures exhibited poor photovoltaic (PV) performance with a planar heterojunction due to the formation of a charge generation layer^37^. Co-evaporation of the two molecules resulted in the formation of a mixed-crystal phase, most likely because of the structural compatibility. Continuous miscibility was found without any phase separation, as typically aimed for in bulk heterojunctions for PV^37^,^38^. The likewise poor performance of the mixed-crystal solar cell was assigned to self-trapping processes.

The present contribution focuses on both the electronic and structural properties of the H16CuPc/F16CuPc heterojunction, and compares planar and bulk types. Planar and bulk heterojunction (PHJ and BHJ, respectively) were prepared on poly(3,4-ethylenedioxythiophene)-poly(styrenesulfonate) (PEDT:PSS) as conductive substrate. The properties of heterojunctions were studied, mostly in a thickness-dependent sequence, with ultraviolet photoelectron spectroscopy (UPS) and angular resolved near-edge X-ray absorption fine structure (NEXAFS) spectroscopy. Details of sample preparation and measurements can be found in the SI [supplementary information]. By combining UPS and NEXAFS results with electrostatic modelling, we provide in the following evidence that (i) E_F_-pinning of one organic layer at the electrode determines the energy level alignment also at the distant organic heterojunction, and that (ii) contact formation induced molecular re-orientation at the organic heterojunction brings about additional filled and empty states at the interface. Both together result in a rather complex energy level landscape including energy level bending across the combined electrode/organic/organic structure, which, however, can fully be rationalized within present state-of-the-art models.

We first discuss the photoemission results for F16CuPc incrementally deposited on H16CuPc and *vice versa*, both planar heterojunctions formed on PEDT:PSS coated indium-tin-oxide on glass as electrode. The valence electron and secondary electron cut-off (SECO) spectra of the pristine H16CuPc and F16CuPc layers are shown as the bottom-most curves in Fig. 3. These layers in direct contact with the electrode are E_F_-pinned^39^,^40^. The reason for this is that the ionization energy (IE) of H16CuPc (4.8 eV) is lower than the work function Φ of PEDT:PSS (4.9 eV), while the electron affinity (EA) of F16CuPc (4.8 eV, using a transport gap of 1.8 eV^41^,^42^ in conjunction with the IE measured here of 6.6 eV) is very similar to Φ of PEDT:PSS. These IE/EA values are compatible with essentially upright standing molecules in the films^27^,^36^.

Upon deposition of the second organic layer on top of the first, the work function of the samples changes gradually and saturates at nominal multilayer coverage (>5 nm); the overall work function change ΔΦ is + 1.0 eV for F16CuPc on H16CuPc (final Φ of 5.4 eV) and −0.6 eV for the reverse sequence with a final Φ of 4.6 eV (Fig. 3a.c). Consequently, some charge density rearrangement must occur across the multilayer structure to change the electrostatic potential. Investigating the valence electron spectra evolution (Fig. 3b,d) upon heterojunction formation, we observe for F16CuPc on H16CuPc that the emission from the respective highest occupied molecular orbital (HOMO) levels shifts *towards* E_F_ for both materials, as emphasized by the dashed lines in the figures. This shift of the first layer is only seen for very low coverage with the second layer due to the high surface sensitivity of UPS. In contrast, for H16CuPc on F16CuPc the emission from the respective HOMO levels shifts *away* from E_F_ for both materials as the heterojunction is formed. Shifts of this sort are in line with energy level bending on both sides of the interface, which necessitates the presence of positive charge carriers within H16CuPc and negative ones in the F16CuPc layer. The driving force for this charge transfer across the organic heterojunction is to be sought in E_F_-pinning of the second layer due to the Φ of the first layer, which is determined through the contact with the electrode. In fact, Φ of the H16CuPc layer E_F_-pinned on PEDT:PSS is as low as 4.4 eV, sufficiently low to enforce E_F_-pinning at the lowest unoccupied molecular orbital (LUMO) level of F16CuPc (EA of 4.8 eV). In analogy – but reversed – Φ of the F16CuPc layer E_F_-pinned on PEDT:PSS is as high as 5.2 eV, high enough to establish E_F_-pinning at the HOMO level of H16CuPc (with an IE of 4.8 eV). In contrast to insulating non-pinned interlayers, as described in the introduction, here both first organic layers are already pinned at E_F_ of the electrode (with opposite charge carrier signs for H16CuPc and F16CuPc, respectively). Therefore, charges from the electrode can further be transferred to the organic/organic interface and accumulate there as required to establish electronic equilibrium. Note that the magnitude of ΔΦ, and thus the amount of charge transferred, at the organic heterojunctions depends on the work function of the first organic layer with respect to the E_F_-pinning level of the second layer. Therefore, ΔΦ is not an intrinsic property of the material combination but it depends on the actual sample-specific work function that is established at the first layer/electrode interface^33^. This situation can be well described within an electrostatic model for the behaviour of energy levels as shown in the SI and explained for general cases in^33^,^43^. Noteworthy is the rather good agreement of our measured pinning Φ values for H16CuPc (4.4 eV on PEDT:PSS and 4.6 eV on F16CuPc/PEDT:PSS) and F16CuPc (5.2 eV on PEDT:PSS and 5.4 eV on H16CuPc/PEDT:PSS) with the corresponding pinning level calculated by Çakir *et al.* from first principles, i.e., 4.41 eV for H16CuPc and 5.21 eV for F16CuPc (both in standing molecular orientation)^20^. While factors like density of gap states due to structural disorder (i.e., sample specific), on-site and inter-molecular Coulomb interaction, and magnitude of energy level bending are not accounted for in these calculations, it might be interesting to explore further theoretical developments towards a more generalized description of organic heterojunctions.

At this point, we have a satisfactory explanation for the observed Φ and energy level changes across the organic heterojunction. To obtain even more insight into the evolution of the HOMO level binding energy as a function of coverage with the second organic layer, we routinely perform a curve fitting procedure that allows disentangling the position of frontier levels more clearly. We use appropriately scaled and shifted spectra of the pristine materials to synthesize a sum spectrum that should ideally match the measured UPS spectrum^44^,^45^. In the attempt to do this for the two interfaces investigated here, we always end up with a residual signal when subtracting the synthesized spectrum from the measured ones, as shown exemplarily in Fig. 4 (more details on this procedure are given in the SI). This *residual signal* can be well simulated as the sum of a H16CuPc and a F16CuPc spectrum, whose energy positions, however, are shifted by an amount ΔIF with respect to those used to construct the *majority* of the measured UPS spectrum; these are labelled as H16CuPc* and F16CuPc* in Fig. 4. Considering the same vacuum level for these contributions as used for the majority signals, we find the IE of H16CuPc* *increased* and that of F16CuPc* *decreased* with respect to those retrieved from the respective thick-film spectra of Fig. 3. Remarkably, the differences in IE values from our analysis match those reported in literature for layers of standing (H16CuPc: 4.8 eV, F16CuPc: 6.6 eV) *versus* lying (H16CuPc: 5.4 eV, F16CuPc: 6.1 eV) molecules^27^. In turn, this suggests that upon heterojunction formation a re-orientation of molecules at the very interface occurs from upright standing to essentially flat lying (whereas second and subsequent layers again exhibit the upright orientation, see above). In contrast to the bulk material, the lying molecules at the interface would not be E_F_-pinned as their respective EA/IE values are well below/above Φ at this stage.

To substantiate the molecular re-orientation at the interface, indirectly inferred from the energy considerations above, we performed angular resolved NEXAFS measurements for the pristine molecular films and a bilayer sample consisting of about 1 nm F16CuPc on top of thick H16CuPc, as shown in Fig. 5a. (For the bilayer sample, the top layer needs to be thin in order to retrieve a signal from both, the F16CuPc and the underlying H16CuPc.) The resulting angular dependence of the C1s → π* transition for the pristine films is shown in Fig. 5b, normalised to the angle of incoming X-rays of 55°^46^, and gives an averaged inclination α of the molecular planes with respect to the substrate surface of about 80°. This is in line with the interpretation from above of essentially upright standing molecules in the pristine films^27^,^36^. For the spectra from the bilayer sample, a linear superposition of the pristine NEXAFS spectra of the two components is formed to separate the angular dependencies of H16CuPc and F16CuPc at the interface. Note that NEXAFS probes X-ray absorption transitions, so that the signal is not affected by the energy level shifts observed in UPS. Since the shifts are caused by electrostatic potential changes all levels are affected in exactly the same way, i.e., transition energies remain constant. The angular dependencies of the components at this interface are displayed in Fig. 5b. It is clearly visible that the molecular orientation at the interface is different compared to that in the pristine layers. Especially the signal from the thin F16CuPc top-layer yields a substantially lower average α of about 30°. Due to the large amount of standing H16CuPc in the bottom layer the change of α is smaller (ca. 10° difference). Nevertheless, the lower α values for the bilayer sample support the coexistence of standing and lying molecules. Taking three standing molecules on the same area as one lying molecule as an estimate, the signal of the bottom-layer H16CuPc comes from two molecular layers of standing (at 80°) and one layer of lying molecules (with 0°). In contrast, the F16CuPc on top would then consist of one layer of lying molecules and ca. 2/3 of a layer of standing molecules (as indicated by UPS, multilayers in the top film exhibit upright orientation). This is in good agreement with the nominal layer thickness of the top layer and the NEXAFS information depth in organic films^47^,^48^.

Consequently, from the combination of NEXAFS and UPS results we have good confidence about the presence of lying molecules at the interface, embedded within the thick layers of standing molecules, as illustrated by the cartoon in Fig. 6. Also possible, but slightly more complex scenarios are discussed in the SI. As to why the re-orientation at the interface occurs, we refer to previous illustration in the following. The preference to form stacks with co-facial and π-overlapping molecules between CuPc and its perfluorinated analogue has been reported before for co-evaporated mixtures^37^,^38^. There, the formation of a mixed-crystal structure was observed without phase separation because of mixed π-stacking. Apparently, the overlap of the π-electron systems of the two different molecules and the reduction of surface energy between the more hydrophilic H16CuPc and the more hydrophobic F16CuPc seems to be the driving force for the behaviour of the blend but also the re-orientation in the present study.

Exposure of molecular surfaces or even interfaces to nitrogen molecules can lead to disorder also in already deposited films^13^,^22^. Also for the deposition of conjugated molecules on top of a molecular film the appearance of disorder^49^ or reorientation^50^ is reported. In the here presented case the structural compatibility of the two involved molecules gives rise for the presented model of co-facial packing at the interface.

Combining the information obtained so far, we can draw the energy diagrams for both layer sequences (Fig. 7), which highlights that both sequences result in the same level alignment for the organic heterojunction. The energy offset between donor and acceptor has to be regarded separately for standing and lying films. The offset between the HOMO levels for lying molecules at the very interface is 0.5 eV, and 1.35 eV for the standing molecules (separated by the lying molecules). The un-pinned bilayer of lying molecules at the interface can be considered as an insulating layer with a linearly changing electrostatic potential, governed by the space charge accumulated on either side in the standing layers.

To complete the analysis of our material combination, the energy levels for co-deposited (BHJ) films of H16CuPc and F16CuPc in a molar ratio of 1:1 were also measured. The evolution of the SECO and the valence spectra are shown in the SI, and the corresponding energy level diagram summarized in Fig. 7c. As representative, the UPS spectrum of a 6.4 nm thick film is shown in Fig. 8. The measured spectrum of the blend can be reproduced by a linear combination of the spectra of the pristine films. Here the adjustment of the spectra with respect to the vacuum level is sufficient to describe the entire blend spectrum over a wide range, as shown. The scaling of the pristine spectra yields a larger amount of F16CuPc at the surface of the film, which also fits the observation of a work function closer to that of the pristine F16CuPc film and the shift to a higher work function with respect to the PEDT:PSS substrate, as seen in Fig. 8a. For this analysis, only standing molecules need to be involved. This is in accordance with previous observations from X-ray scattering of blend films^37^. The HOMO level offset between donor and acceptor in the blend is 0.6 eV. Note that this is in good agreement with the HOMO level offset found for lying molecules in the planar heterojunction, where the π-π interaction of the two molecules is expected to be similar to the mixed crystal.

The present analysis of the interface between H16CuPc and F16CuPc was also motivated to better understand the behaviour of photovoltaic cells containing these materials as donor and acceptor, and their function as charge generation layer. A new quality in the interpretation of the interface between these two molecules is gained from the combined analysis of morphology and energy levels at the direct interface. The photovoltaic gap (E_PVG_) as difference between the HOMO level onset of the donor and the LUMO level onset of the acceptor determines the upper limit of the open circuit voltage in organic solar cells^3^,^51^,^52^,^53^,^54^,^55^. Using a transport gap of 1.8 eV for both phthalocyanines^41^,^42^ and both orientations^15^,^56^, E_PVG_ is 1.3 eV for the very interface between the lying molecules. Due to energy level bending and the different IE, E_PVG_ is reduced to 0.45 eV between the standing molecules (separated by the lying molecules, ca. bilayer). The bulk heterojunction gives a value of E_PVG_ = 1.2 eV. In a previous report, the PHJ devices did not exhibit any response to light^37^, despite the fact that the generation of electrons and holes at the interface seems possible from the energy level diagram (Fig. 7). However, holes and electrons generated at the interface or close to the interface have to overcome a barrier due to the energy level bending when moving away. This energy step seems to suppress the transport of holes and electrons away from the interface and therefore the photovoltaic response. Besides the absent photovoltaic effect, the interface acts as a charge generation layer in planar heterojunction devices^37^. Therefore, a low energy gap between the LUMO level onset of the acceptor and the HOMO level onset of the donor is necessary to allow for the tunnelling of electrons from the HOMO of the donor to the LUMO of the acceptor under reverse bias^57^. The HOMO-LUMO level gap between the standing molecules of the two Pc’s is indeed only 0.45 eV; even with the lying molecule bilayer as additional barrier the tunnelling process occurs in the reported devices^37^. Also the before mentioned extraction barrier can now be overcome due to the applied reverse bias. This is in contrast to forward biasing a diode where the charges are injected at the electrodes and the transport occurs towards the interface.

The open circuit voltage of a bulk heterojunction solar cell made from the molecules investigated here is about 0.35 V^37^. This is only half of the expected value from E_PVG_ considerations, reduced by a general loss observed for planar heterojunction cells of about 0.5 eV^54^. Accordingly, the short circuit current is roughly two orders of magnitude lower than what is typically found for organic photovoltaic cells^37^. The absence of phase separation between the two constituents leads to a very low probability for the charge carriers to find percolation paths to the respective electrodes^37^, resulting in massive recombination losses of charge carriers in the charge extraction process.

In summary, the combined analysis of the electronic structure and morphology at the F16CuPc/H16CuPc interface leads to a clear-cut interpretation of the corresponding electrical behaviour in devices. In a planar heterojunction, the molecules form a bimolecular interlayer at the interface with a co-facial, π-orbital overlap between the two different species. In this interlayer, the molecules are oriented mainly parallel to the substrate surface whereas the surrounding (bulk) molecules are standing upright. The description of the interface region as a combination of standing and lying molecules of both species leads to the comprehensive picture of the energy levels across the heterostructure. The films formed by standing molecules at the PHJ are Fermi-level pinned resulting in a charge transfer from the substrate to the organic/organic interface, sheet charge densities at both sides of this interface, and energy level bending close to the interface. In contrast, the lying molecules in the interlayer are unpinned and undergo a linear change of the electrostatic potential. The blend films can be described by standing molecules in agreement with the morphological data from X-ray scattering^37^. The observed energy landscapes assist the understanding of the diode and solar cell behaviour of this material pair reported in the literature^37^. The observed π-overlap between different molecules in the interlayer may also be of interest within the context of molecular hybridisation^58^,^59^, photovoltaic active interfaces^60^,^61^, or the analysis of (dynamic) charge-transfer states^62^,^63^ in follow-up studies. In all cases, the reliable correlation of interface morphology and structure with the resulting electronic properties is the necessary starting point for deriving comprehensive structure-property-function relationships.

## Additional Information

**How to cite this article**: Opitz, A. *et al.* Organic heterojunctions: Contact-induced molecular reorientation, interface states, and charge re-distribution. *Sci. Rep.* **6**, 21291; doi: 10.1038/srep21291 (2016).

## Supplementary Material

Supplementary Information

## Figures and Tables

**Figure 1:**
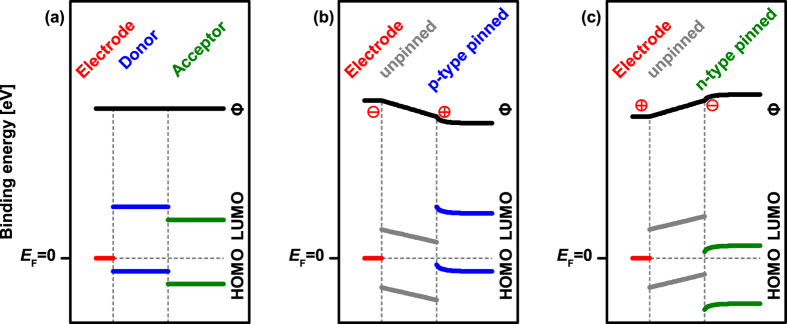
Schematic energy level diagrams for organic/organic heterojunctions with a bottom electrode. (**a**) In the case of vacuum level alignment at the electrode/organic and at the organic/organic interface the electrostatic potential ϕ (equivalent to work function) is constant. (**b,c**) Fermi level pinning occurs for both charge carrier types across an interlayer of unpinned material and results in a linear change of the electrostatic potential ϕ in the unpinned materials and shows energy level bending inside the pinned material. Charge transfer is indicated by the plus and minus signs for the respective areas where the sheet charge density is present.

**Figure 2:**
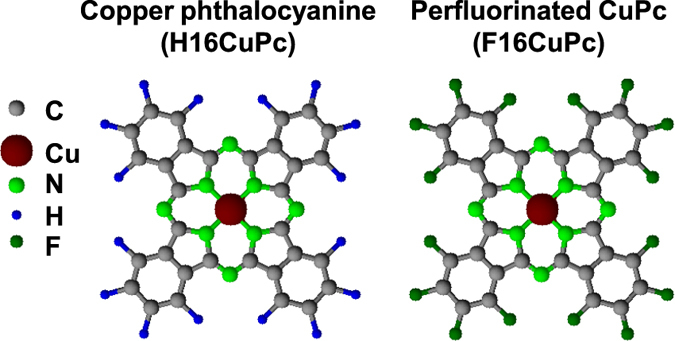
Chemical structures of hydrogen-terminated copper phthalocyanine H16CuPc and its perfluorinated analogue F16CuPc.

**Figure 3:**
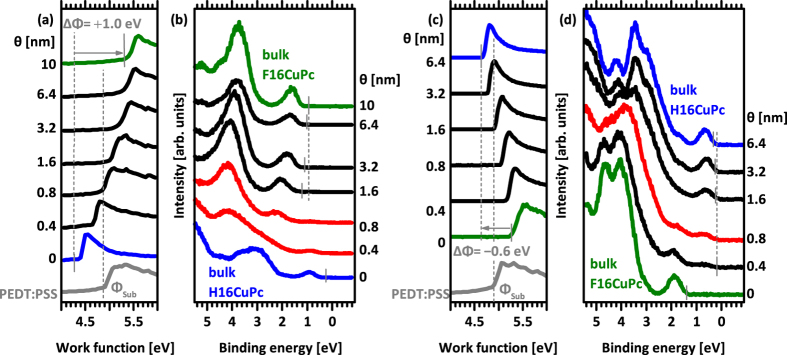
Secondary electron cut-off (SECO) and valence region photoemission spectra for both layer sequences of PHJ with increasing thickness of the top layer. (**a**) SECO and (**b**) valence region for F16CuPc molecules deposited on top of H16CuPc film. (**c**) SECO and (**d**) valence region spectra for H16CuPc molecules deposited on top of F16CuPc film. Additionally, the SECO of the underlying PEDT:PSS electrode is shown. Measurements were done with illumination from a He discharge lamp (a + b) and synchrotron radiation (c + d), which caused differences of the bulk spectra in (**b,d**).

**Figure 4:**
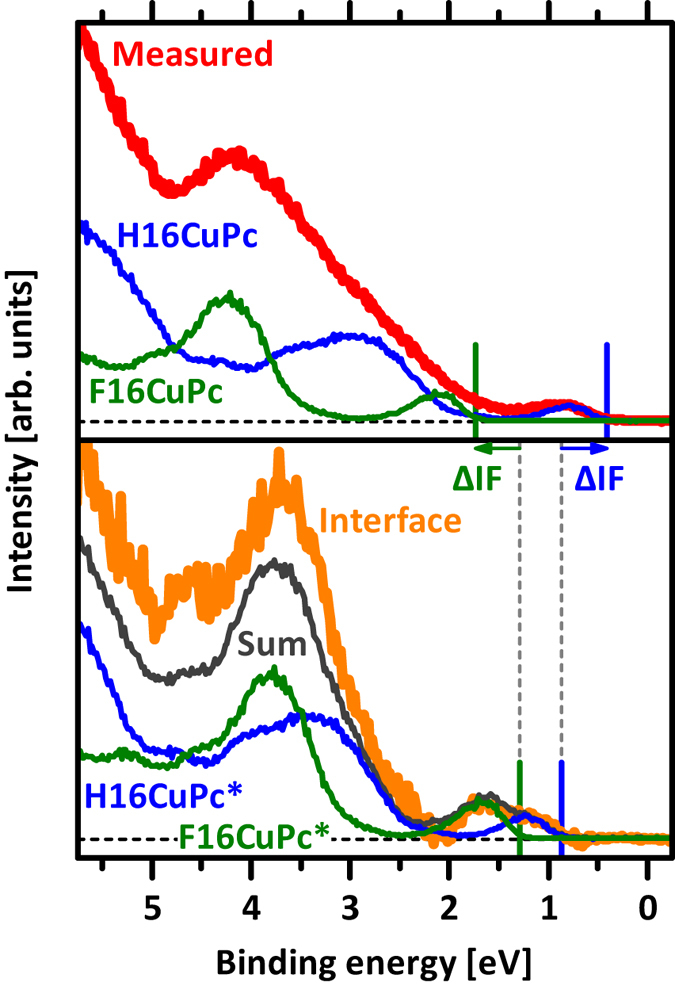
Deconvolution of the measured UPS spectrum with 0.4 nm F16CuPc on top of an H16CuPc film. Subtraction of the scaled spectra of the pristine films (H16CuPc and F16CuPc, upper diagram) from this measurement results in the interface spectrum (lower diagram), which can be described by two additional components of H16CuPc* and F16CuPc* with spectral shifts of the onsets (ΔIF) for both components with respect to the components of the pristine films. The superposition of the scaled and shifted spectra is shown as sum.

**Figure 5:**
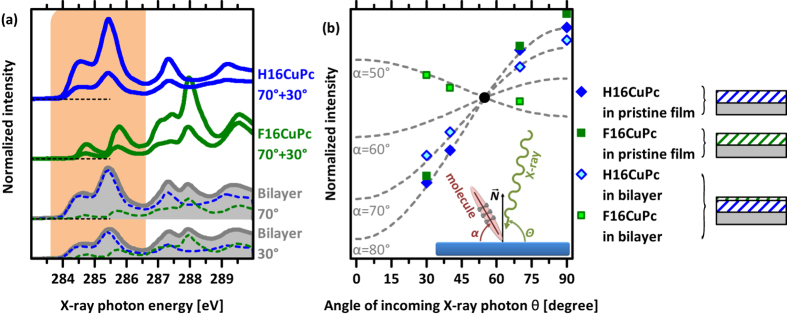
(**a**) Normalized NEXAFS spectra for pristine films of H16CuPc and F16CuPc and for the interface of a thin film of F16CuPc on top of H16CuPc for angles of incoming X-ray beam at 70° and 30°. The deconvolution into the spectra of the pristine materials is shown for the respective spectra of the two layer system (dashed lines). The marked energy range between 283.6 and 286.6 eV is used for the analysis of angular dependence and of spectral superposition. (**b**) Angular dependence of NEXAFS signals of pristine films and individual components of interfacial bilayer together with simulated dependencies^46^ for given molecular angles normalised to the angle of incoming X-ray photons of 55°. The inset shows the NEXAFS geometry.

**Figure 6:**
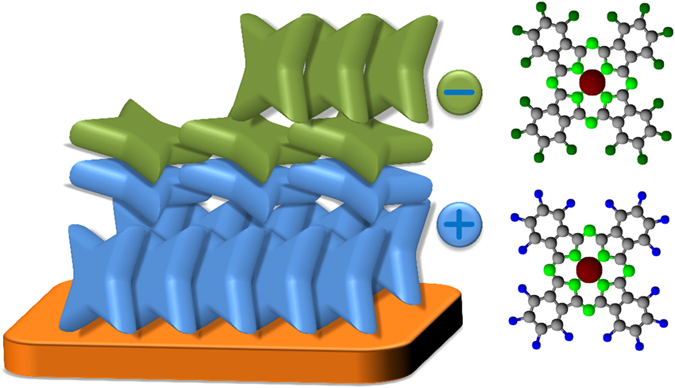
Schematic molecular arrangements for the co-facial interlayer at the interface of a planar heterojunction between films of H16CuPc (lower layer) and F16CuPc (upper layer). The plus and minus signs indicate the sheet charge density at the organic/organic interface. The discussion of other possible molecular arrangements is given in the SI.

**Figure 7:**
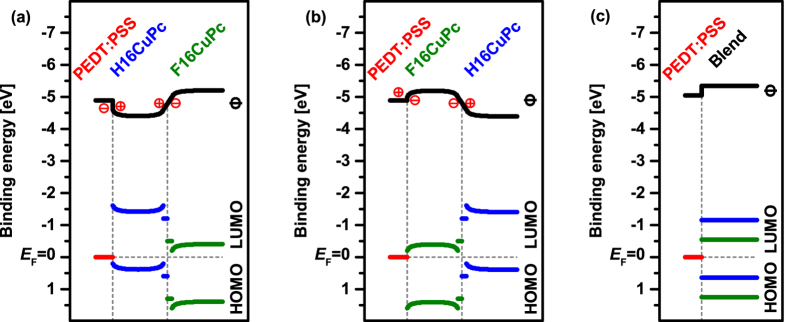
Energy level diagram for planar heterojunctions with both layer sequences of phthalocyanine molecules: (**a**) F16CuPc/H16CuPc, (**b**) H16CuPc/F16CuPc, and (**c**) for the blended film, all deposited on PEDT:PSS. The horizontal dashed line gives the Fermi-level for the layer stack, the vertical dashed line marks the interfaces between the different materials. Charge transfer at planar interfaces is indicated by the plus and minus signs.

**Figure 8:**
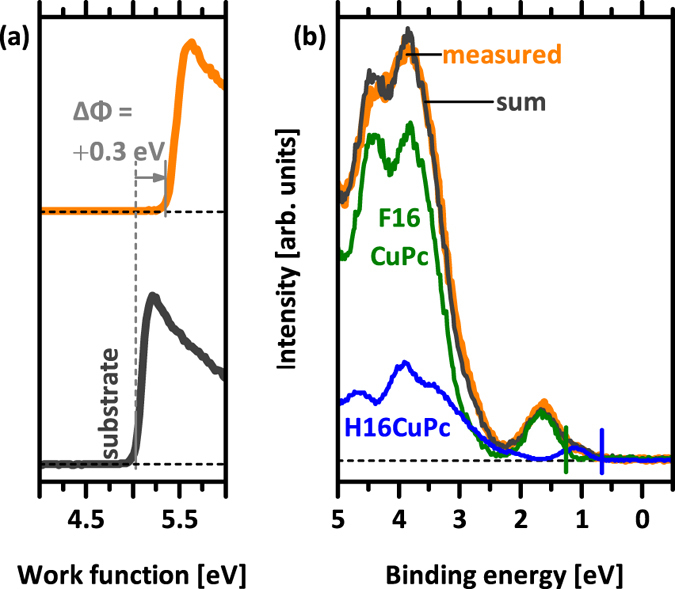
(**a**) SECO and (**b**) valence region spectra for a blended film of H16CuPc and F16CuPc with a thickness of about 6.4 nm and a nominal blend ratio of 1:1. The superposition (sum) is realised by taking scaled spectra of pristine materials to describe the blend spectrum. Measurements are done with illumination from synchrotron radiation.
