# Supplementary material for: Organic heterojunctions: Contact-induced molecular reorientation, interface states, and charge re-distribution
Source: Sci Rep. 2016 Feb 18;6:21291. doi: 10.1038/srep21291 (PMC4758050; doi:10.1038/srep21291)
Supplement: Supplementary Information [file srep21291-s1.pdf]

Electronic supplementary information (ESI) for

Organic heterojunctions: Contact-induced molecular reorientation, interface states, and charge re-distribution

Andreas Opitz[(1)]*, Andreas Wilke[(1)], Patrick Amsalem[(1)], Martin Oehzelt[(1,2)], Ralf-Peter Blum[(1)], Jürgen P. Rabe[(1)], Toshiko Mizokuro[(3)], Ulrich Hörmann[(4)], Rickard Hansson[(5)], Ellen Moons[(5)], Norbert Koch[(1,2)]*

(1) Institut für Physik & IRIS Adlershof, Humboldt-Universität zu Berlin, Berlin, Germany

(2) Helmholtz-Zentrum Berlin für Materialien und Energie GmbH, Bereich Erneuerbare Energien, Berlin, Germany

(3) National Institute of Advanced Industrial Science and Technology, Osaka, Japan

(4) Institute of Physics, University of Augsburg, Augsburg, Germany

(5) Department of Engineering and Physics, Karlstad University, Karlstad, Sweden

*Contact: Andreas.Opitz@hu-berlin.de, Norbert.Koch@physik.hu-berlin.de

---

(1) Experimental

Solvent cleaned indium-tin oxide coated glass substrates or silicon wafers covered with native oxide were treated with UV/ozone to create a hydrophilic surface. Poly(3,4-ethylenedioxythiophene)-poly(styrenesulfonate) (PEDT:PSS, purchased as AI4083 from H.C. Starck Gmbh & Co. KG) was spin coated on top to form a high work function electrode. The spin coated PEDT:PSS was then annealed at 180°C for 10 min under ambient conditions. These substrates served as templates for vacuum deposition of the organic semiconductors copper phthalocyanine (H16CuPc) and its perfluorinated version F16CuPc (both purchased from Sigma Aldrich and used without further purification). The evaporation of the organic semiconductors was carried out in UHV using resistively heated pinhole sources with evaporation rates of about 0.1 nm/min. The mixed layer of H16CuPc and F16CuPc was grown by co-deposition from two spatially separated evaporation sources. The thicknesses of the molecular layers were monitored using a microbalance therefore every given layer thickness in this work corresponds to a nominal mass-thickness. The samples were prepared in a preparation chamber with a base pressure of $<6\times10^{-8}$ mbar. The analysis chamber (base pressure 1×10^{-10} mbar) was directly connected to the preparation chamber, enabling sample transfer without breaking vacuum. Ultraviolet photoelectron spectroscopy (UPS) measurements were carried out at the synchrotron radiation source BESSY II (Berlin, Germany) with a hemispherical electron analyser (Scienta SES 100) or were performed in-house using a He discharge lamp (He I of 21.22 eV and He II of 40.8 eV) and a Phoibos 100 (Specs) hemispherical energy analyser. The secondary electron cut-offs (SECOs) were measured with the samples biased at –10 V. Angular resolved near-edge X-ray absorption fine structure (NEXAFS) spectra were recorded on ex-situ prepared samples at MaxLAB synchrotron facility (Lund, Sweden) by detecting the partial and the total electron yield (PEY, TEY) using multi-channel plate detector and current meter, respectively. Spectra were taken in the energy range from 276 eV to 327 eV and also normalized in this range. To ensure that differently

oriented transition dipole moments of the molecules do not interfere, the analysis of angular dependence and of spectral superposition was done in the range from 283.6 eV to 286.6 eV. This energy range corresponds to electronic transitions from the C1s core level to the π* molecular orbital [1]. While the data is shown for the TEY signal only, similar results are observed for the PEY signal.

(2) Deconvolution of interface spectra

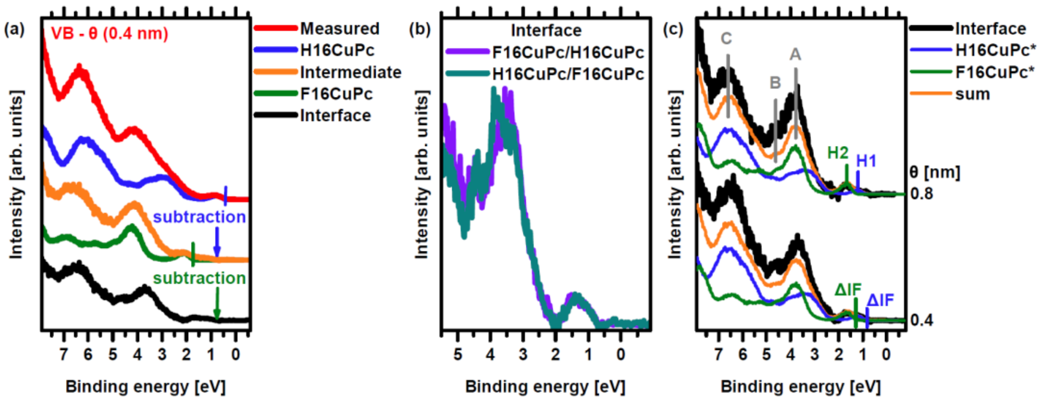

Supplementary figure S1: (a) Valence region spectra for 0.4 nm F16CuPc on top of H16CuPc—taken from Figure 3b. The interfacial valance region spectrum is determined by stepwise subtraction of the scaled spectra taken from pristine materials without energy shift with respect to the vacuum level of this certain measurement. (b) Comparison of interface spectra for the different layer sequences. (c) Decomposition of interface spectra for two different film thicknesses of F16CuPc on top of H16CuPc based on the spectra of the pristine materials allowing also an energy shift at the interface ΔIF in comparison to the onset values used in (a).

Supplementary figure S1a shows the stepwise subtraction of the pristine film spectra from the measured UPS spectrum to determine the interface spectrum for coverage of 0.4 nm F16CuPc on top of an H16CuPc film. The interface spectra for both layer sequences (F16CuPc on top of H16CuPc and vice versa) are within the error of the measurement equivalent as illustrated in Supplementary figure S1b. Spectra of the pristine materials are used with a shift in the binding energy to further deconvolute the interface spectra. The linear superposition of the pristine spectra for both components and the interface spectra are displayed in Supplementary figure S1c. The pristine spectra have to be shifted in binding energy by +0.4 eV and 0.5 eV for H16CuPc and F16CuPc, respectively. When intensity and energetic positions are adjusted to match features H1 and H2, the shape of the interface spectrum is well reproduced (peaks A, B, C), except for a general mismatch in intensity. This is a clear hint that no new states are formed by the molecular interaction at the interface.

(3) Electrostatic simulations

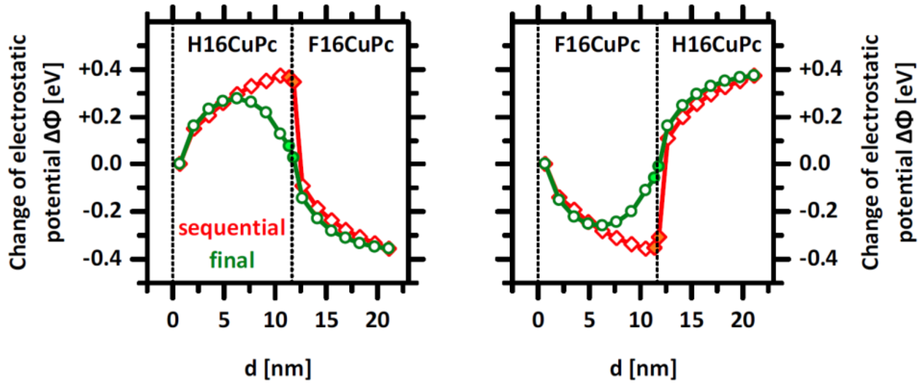

Supplementary figure S2: Calculations [2] of the change of electrostatic potential at the surface for sequential produced layer stacks (red diamonds) which reproduces the shown measurements together with the electrostatic potential inside the final stacks (green circles). Each final stack consists of the co-facial double layer of lying molecules at the interface (indicated by the vertical dashed line at the right) which is sandwich between 7 layers of standing molecules at both sides deposited on the substrate located at thickness 0 nm (indicated by the vertical dashed line at the left). The layers with changed molecular orientation at the interface are visualised with filled symbols.

Electrostatic calculations [2] were performed for the presented heterojunction system in dependence on the layer thickness as displayed in Supplementary figure S2. The energy levels therefore are given in the paper. Additional parameters are the width of the Gaussian density of states with 0.25 eV and the substrate work function of 4.8 eV for both cases. The difference between the values according to the experiment (red diamonds) for each layer and the values inside the final stack is largest at the organic/organic interface.

(4) Thickness series for mixed film

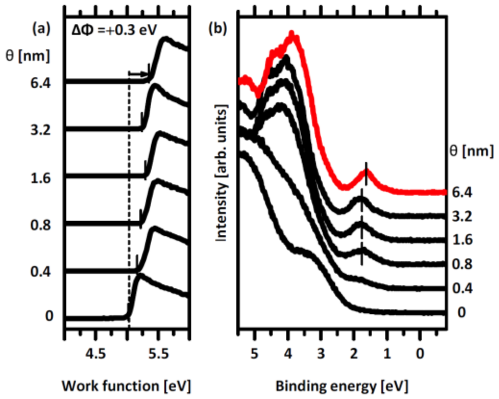

Supplementary figure S3: Thickness series with (a) SECO and (b) valence region spectra for blend of H16CuPc and F16CuPc with a nominal blend ratio of 1:1. Measurements are done with illumination from synchrotron radiation.

The energy levels were measured for mixed films of H16CuPc and F16CuPc in dependence on the film thickness deposited on top of PEDT:PSS at a nominal mixing ratio of about 1:1. The evolution of the SECO and the valance region are shown in Supplementary figure S3a+b, respectively. An increase of the work function with increasing thickness is observed up to a value of 5.3 eV which is very close to the value of pristine F16CuPc on such substrates. No observable shift of the valence band spectra can be seen for the deposition up to 3.2 nm of the blend. A rigid shift was found between a blend film thickness of 3.2 nm and 6.4 nm.

(5) Molecular arrangement at interface and in blend

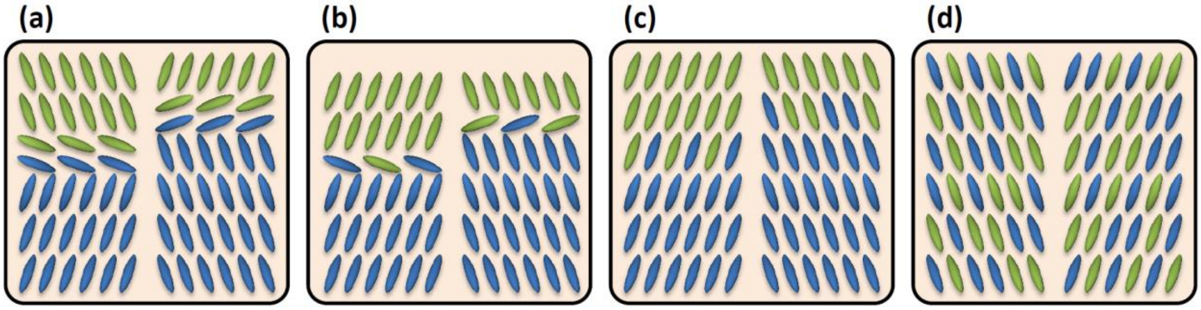

Supplementary figure S4: Molecular arrangements for the interface (a-c) and the blend (d).

Different molecular arrangements for the molecules at the planar interface are possible. Supplementary figure S4a recapitulates the model shown in the manuscript. Here clear phase separation of H16CuPc and F16CuPc is assumed. Intermixed interlayer structures are shown Supplementary figure S4b+c. Lying molecules are present in model shown in Supplementary figure S4b. From energetically point of view, a single layer with lying molecules of different Pc molecules is very unlikely. π-orbital overlap is one of the driving forces for morphological arrangements. In contrast, a mixed layer of standing molecules is able to happen due to interdiffusion (see Supplementary figure S4c), but can be suspend by our presented measurements. The structure for the blend is adapted from Reference [3].

References

[1] J. Stöhr, D. Outka, Determination of molecular orientations on surfaces from the angular dependence of near-edge x-ray-absorption fine-structure spectra, Phys. Rev. B. 36 (1987) 7891–7905. doi:10.1103/PhysRevB.36.7891.

[2] M. Oehzelt, N. Koch, G. Heimel, Organic semiconductor density of states controls the energy level alignment at electrode interfaces, Nat. Commun. 5 (2014) 4174. doi:10.1038/ncomms5174.

[3] A. Opitz, B. Ecker, J. Wagner, A. Hinderhofer, F. Schreiber, J. Manara, et al., Mixed crystalline films of co-evaporated hydrogen- and fluorine-terminated phthalocyanines and their application in photovoltaic devices, Org. Electron. 10 (2009) 1259–1267. doi:10.1016/j.orgel.2009.07.004.
